# The Selective Cyclooxygenase-2 Inhibitor, the Compound 11b Improves Haloperidol Induced Catatonia by Enhancing the Striatum Dopaminergic Neurotransmission 

**Published:** 2010

**Authors:** Hadi Fathi-Moghaddam, Mehdi Shafiee Ardestani, Mostafa Saffari, Ali Jabbari Arabzadeh, Mitra Elmi

**Affiliations:** a *Depattment of Physiology and Physiology Research Center, School of Medicine, Jondishapour University of Medical Sciences, Ahwaz, Iran. *; b *Department of Pharmacology, Razi institute for drug research, College of Medicine, Iran University of Medical Sciences, Tehran, Iran. *; c *Department of Medicinal Chemistry and Radiopharmacy, Faculty of Pharmacy, Tehran University of Medical Sciences, Tehran, Iran*.; d *Department of Pharmaceutics, Faculty of Pharmacy, Shahid Beheshti University of Medical Sciences, Tehran, Iran. *

**Keywords:** Catalepsy, Nigrostriatal, Compound 11b, Dopaminergic neurotransmission

## Abstract

A substantial amount of evidence has proposed an important role for Cyclooxygenase-2 (COX-2) enzyme in brain diseases and affiliate disorders. The purpose of this research was studying the effects of COX-2 selective inhibition on haloperidol-induced catatonia in an animal model of drug overdose and Parkinson’s disease (PD). In this study, the effect of acute and Sub-chronic oral administration of a new selective COX-2 inhibitor, i.e. the compound 11b or 1-(Phenyl)-5-(4-methylsulfonylphenyl)-2-ethylthioimidazole, in a dosage of 2, 4 and 8 mg/kg on haloperidol-induced catatonia was evaluated and compared to the standard drug scopolamine (1 mg/kg) by microanalysis of Striatum dopaminergic neurotransmission. The results showed a very high potency for 11b in improving the catalepsy by enhancing the dopaminergic neurotranmission (p < 0.05). In addition, statistical analysis showed the dose- and time-dependent behavior of the observed protective effect of 11b against the haloperidol-induced catatonia and enhancement of the dopaminergic neurotransmission. These findings are additional pharmacological data that suggest the effectiveness of COX-2 inhibition in treatment of schizophreny-associated rigidity.

## Introduction

Antipsychotics, which are routinely used in management of schizophrenia and other related disorders, are often associated with distressing extra-pyramidal side effects. The cataleptic immobility induced by typical neuroleptics (e.g., haloperidol) in animals is a good behavioral model to study the nigrostriatal function and its modulation by cholinergic, dopaminergic, and other systems (1). Haloperidol-induced catalepsy occurs due to the blockade of dopamine (D^2^) receptors and reduced dopaminergic neurotransmission. Enhanced stimulation of the central cholinergic system is also involved in haloperidol-induced catalepsy as this catalepsy has been reported to be enhanced by pilocarpine and antagonized by the cholinergic blocker atropine (1).

Interesting evidence suggests that besides the more important roles for inflammatory reactions, cyclooxygenase-2 (COX-2) causes some pathological processes which are seen in many neurodegenerative disorders including Parkinson’s disease (PD) (2, 3). Furthermore, inhibition of COX-2 or COX-2 gene expression has been shown to improve the movement disorders of PD in animal models (4, 5). Also previous reports suggest that COX-2 could increase the acetylcholine level in brain by producing the prostaglandin E2 and increasing the expression of cholinergic important factors such as choline acetyl transferase and vesicular acetylcholine transporter protein. It has also been suggested that prostaglandins have modulatory effects on adrenergic, noradrenergic and glutaminergic neurotransmission (6, 7). In addition, some of the investigations have shown that COX-2 inhibitors impair the spatial memory through reduction of acetylcholine level in brain (8, 9). 

The compound 11b, *i.e*. 1-(Phenyl)-5-(4-methylsulfonylphenyl)-2-ethylthioimidazole, was used to find a relationship between striatum dopaminergic neurotransmission changes and drug induced catalepsy as PD and neuroleptic overdosage animal models after selective COX-2 inhibition. This compound has previously been described as the most potent and selective COX-2 inhibitor (COX-2 IC_50_ = 0.58 μM with no inhibition of COX-1 up to 25 μM) relative to the reference drug celecoxib (COX-2 IC_50_ = 0.21 μM with no inhibition of COX-1 up to 25 μM) (10). 

## Experimental

*Animals*

Adult male albino rats (weighing 250-300 g) were selected for the study. The animals were purchased from Pasteur Institute of Iran and housed in stainless steel cages, handled daily, and provided with food and water *ad libitum*. A 12 h light/12 h dark cycle was maintained, and the animals were tested during the light cycle. These animal experiments were carried out in accordance with recommendations of the declaration of Helsinki, and the internationally accepted principles of using experimental animals. 

*Chemicals*

The compound 11b was prepared by a method based on a previously established procedure (10). Scopolamine and haloperidol were purchased from Merck (Germany). Compound 11b and haloperidol were dissolved in distilled water, and scopolamine was dissolved-suspend in 1% Gum acacia solution. In acute studies all injections were IP, while in chronic studies all injections were PO except for haloperidol which was IP. 

*Surgery and Microdialysis procedure*

The rats were first anesthetized by 75 mg/kg of ketamine combined with 8 mg/kg of Xylazin IP (4, 5), and then placed in the stereotaxic apparatus and a sagittal incision was made in the scalp with sterile blade. The skin and inferior tissue layers covering the skull were retracted to make the skull exposed after which a hole was drilled through the skull in the area overlaying the right striatum, using the following coordinates with respect to the bregma: A/P + 1 mm; M/L + 3 mm, D/V + 6 mm according to the atlas (11). A guide-cannula was lowered into the brain for inserting the microdialysis probe delivering a modified Ringer solution, and was fixed to the cranium after which the incision was closed. Surgery was performed using sterile instruments and aseptic conditions. Rats were allowed to recover from the surgery for 7–10 days. On the experimental day, a microdialysis probe was inserted into the cannula, and the input sides of the probes were connected to a microperfusion pump, *i.e. *CMA/102 infusion pump (CMA/Microdialysis, Sweden), which delivered a modified Ringer solution (147 mM NaCl, 1.2 mM CaCl_2_, 2.7 mM KCl, 1.0 mM MgCl_2_ and 0.04 mM ascorbic acid) at a flow rate of 2 μL/min. Ringer solution was then infused for 3 to 3.5 h before the baseline samples were collected to obtain stable basal extracellular levels of dopamine. The microdialysate samples (20 μL) were then collected every 20 min (4). When a stable outflow was shown by four consecutive samples of neurotransmitters, rats were orally given the compound 11b (2, 4 and 8 mg/kg) and scopolamine (1 mg/kg) using Dimethyl Sulfoxide (DMSO) as the vehicle. Control rats received a saline injection (1 mL/kg). The dialysates were collected for 4 h after the administration of drug-vehicle samples. The stress caused by the IP vehicle injection and handling of the rats was found not to alter the extracellular glutamate-dopamine levels. In some experiments, when the rats were given drugs or vehicle after four stable consecutive samples, the dialysates were collected for 2.5 h after the injection. Microdialysate dopamine levels were analyzed immediately. After the experiments, the histological locations of the probes were determined on serial coronal sections. Only data obtained from the rats with correctly implanted probes were included in the results. All experiments were made with conscious animals. Animals were individually housed for the duration of the experiment in a CMA/120 system (CMA/Microdialysis, Sweden). This procedure was performed at the first and seventh days of the study. 

Dopamine samples were analyzed by reverse-phase High Performance Liquid Chromatography (HPLC) with electrochemical detection (4, 12). 

*Experimental procedure*

Concurrently to the microdiaysis procedure, catatonia was induced with haloperidol (1 mg/kg) and assessed at 30 min intervals until 120 min and then at 240 min, by means of standard bar test (13, 14). Haloperidol (1 mg/kg IP) was chosen since it could induce a moderate degree of catalepsy, thus enabling the detection of either attenuation or potentiation (15, 16). Catalepsy was assessed in terms of the times for which the rats maintained an imposed position with both front limbs extended and resting on a 10 cm high wooden bar (1.25 cm diameter). The end point of catalepsy was considered to be the time when both front paws were removed from the bar or when the animal moved its head in an exploratory manner. A cut-off time of 300 sec was applied. Between the determinations, the animals were returned to their individual home cages. 

Scoring method: If the animal maintained the imposed posture for at least 20 sec, it was considered to be cataleptic and given one point. For every additional 20 sec that the cataleptic posture was maintained, one extra point was given. The animals were tested twice at 30 min intervals and only the greater duration of immobility was considered. In the acute studies, the compound 11b (2, 4 and 8 mg/kg), or scopolamine (1.0 mg/kg) were administered only once at 30 min before the haloperidol administration. In the Sub-chronic studies, these agents were administered once daily 30 min prior to the haloperidol administration for seven days. Catalepsy was determined 30 min after haloperidol administration on the first and seventh days of treatment.

*Statistical analysis*

*Catalepsy*

For each group, the data were shown as mean ± SEM and analyzed by one way analysis of variance (ANOVA) followed by Dunnet Multiple Comparision test, and P values less than 0.05 were considered to be statistically significant.

*Microdialysis*

The average concentration of four stable samples before drugs or vehicle (DMSO) administration was considered as the basal glutamate-dopamine concentration. Statistical evaluation of the results was performed by means of one-way analysis of variance (ANOVA) and Student–Newman–Keuls multiple range test, considering the p*-*values less than 0.05 to be statistically significant.

## Results

*Acute study*

**Figure 1:**
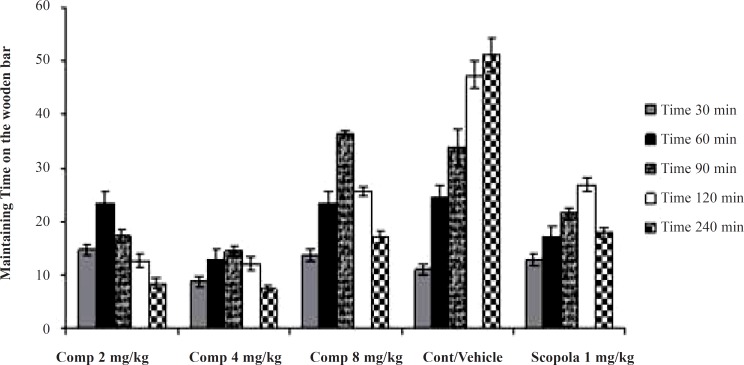
The effects of acute administration of the compound 11b as a new selective COX-2 inhibitor on haloperidol- induced catalepsy

In the acute studies, administration of the standard drug (scopolamine) and all doses of the test drug gave cataleptic scores similar to the vehicle-treated groups. However, from 60 min onwards after haloperidol administration, scopolamine (1.0 mg/kg) resulted in significantly lower cataleptic scores than those in the vehicle-treated rats. On the other hand, the median dose and the lower dose of the compound 11b resulted in lower cataleptic scores as compared to the vehicle-group 60 and 90 min after haloperidol administration, respectively. However, from 120 min until 240 min after haloperidol administration, the higher dose of the compound 11b significantly lowered the cataleptic scores compared to the vehicle group. In fact, 90 min post-haloperidol, all doses of the compound 11b were actually more protective against catalepsy even compared to the standard drug scopolamine. Surprisingly, the higher dose of the compound 11b was observed to be less effective in catalepsy improvement compared to other doses (Figure 1). 

*Sub-chronic study*

**Figure 2:**
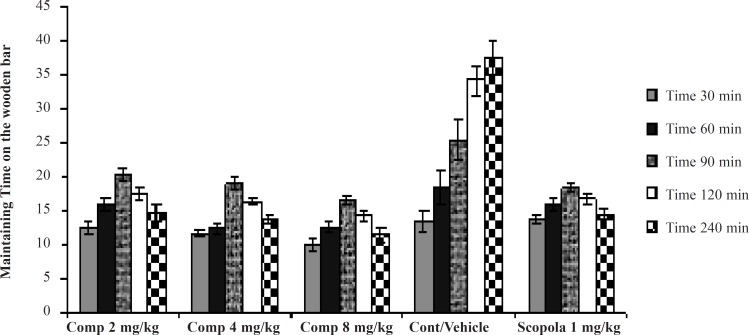
The effects of Sub-Chronic administration of the compound 11b as a new selective COX-2 inhibitor on haloperidol- induced catalepsy

In the Sub-chronic studies, administration of the standard drug and all doses of the test drug 30 min after the last haloperidol dose on the seventh day, gave cataleptic scores similar to the vehicle-treated groups. However, from 60 min onwards, scopolamine (1.0 mg/kg) and the compound 11b (2, 4 and 8 mg/kg) resulted in significantly lower cataleptic scores than the vehicle-treated rats. These results showed that the highest dose of the compound 11b was as protective as the 1 mg/kg dose of scopolamine. Our statistical analysis showed that the protective effect of the compound 11b against haloperidol-induced catatonia was both dose- and time- dependent (Figure 2). 

*Acute Microdialysis Study*

The striatal extraneuronal (*i.e*. microdialysate) concentration of dopamine in rats of all groups before drug-vehicle injection (baseline) was 2.1 ± 0.33 pg/20 μL.

**Figure 3:**
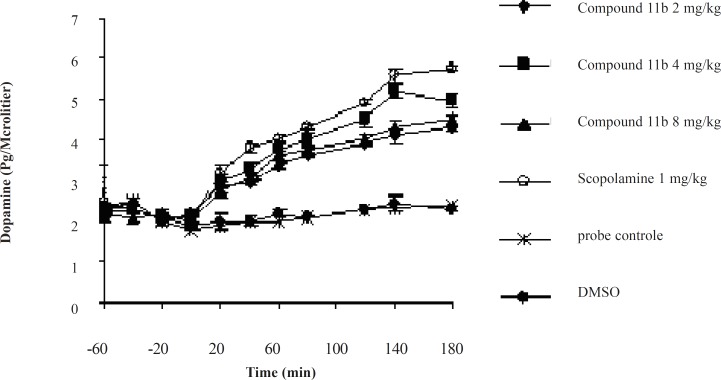
The effects of acute administration of the compound 11b as a new selective COX-2 inhibitor on Striatum dopaminergic neurotransmission

The different doses of COX-2 inhibitor and scopolamine were shown to modify the dopaminergic neurotransmission in the striatum of rats during the observation periods. Additionally, the changes were proved to be significant for dopamine concentrations (p < 0.05) on or after 40 min throughout the 180 min period (Figure 3). 

*Sub-chronic Microdialysis Study*

**Figure 4:**
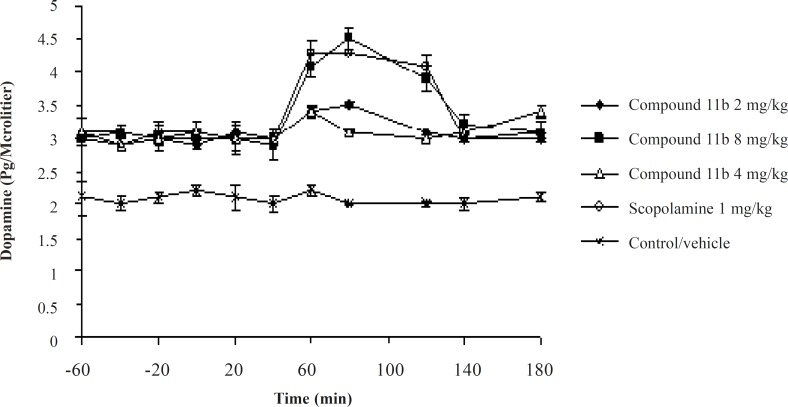
The effects of Sub-Chronic administration of the compound 11b as a new selective COX-2 inhibitor on Striatum dopaminergic neurotransmission

The striatal extraneuronal (*i.e*. microdialysate) concentration of dopamine in rats of all groups before drug-vehicle injection (baseline) was 2.9 ± 0.41 pg/20 μL. This observation shows that the sub-chronic administration of the selective COX-2 inhibitor, the compound 11b, could elevate the basal level of Striatum dopamine from 2.1 ± 0.33 pg/20 μL to 2.8 ± 0.41 pg/20 μL. The seventh day results of the microdialysis experiment showed a significant increase in the striatal dopaminergic neurotransmission compared to the seventh day basal level after administrantion of the selective COX-2 inhibitor at its high dose of 8 mg/kg and scopolamine at a dose of 1 mg/kg during the 40-120 min period (Figure 4).

## Discussion

This study was intended to investigate the more important role for COX-2 in dopaminergic neurotransmission in striatum and also the haloperidol-induced catalepsy. 

It seems that COX-2 and its major products, *i.e*. prostaglandins (PGs), have an important role in neurotransmitter release and PD-associated rigidity as reported in previous works (4, 5, 17, 22), suggesting that COX-2 causes increased levels of acetylcholine in brain via the production of PGE2 and increases in expression of cholinergic markers, such as choline acetyl transferase and vesicular acetylcholine transporter protein. It was also noted that prostaglandins and specially PGE2, had modulatory effects on adrenergic, noradrenergic and glutaminergic neurotransmission, and prostaglandin synthesis inhibitors induced an increase in blood pressure via an increase in catecholamine release; for example using large doses of glucocorticoids in human may cause insomnia, euphoria and an increase in intracranial pressure. Additionally, an *in-vitro *study evaluated the effects of some NSAIDs on cultured primary rat embryonic neurons from rat embryos mesencephalon also containing glial cells (an experimental preparation that reflected the cellular composition of the brain, thus useful in studying neuroinflammation). Incubation with aspirin, paracetamol or ibuprofen protected dopaminergic neurons from glutamate toxicity. The study considered the reduction of the decrease in dopaminergic uptake caused by glutamate, and the attenuation of the tyrosine hydroxylase-positive cell loss as indices. Among the NSAIDs tested, ibuprofen was the most effective and surprisingly increased the number of dopaminergic cells in basal condition most likely by protecting them from the excitotoxicity associated with culture medium change. An interesting report from a chronic observation was that haloperidol and chlorpromazine significantly decreased the dopamine, norepinephrine and serotonin levels in brain homogenates while causing a significant increase in metabolites’ levels (VMA and HVA) in urine (18). This observation was from a chronic study performed over a 21 days period, and it is expected to find a decreased level in free dopamine and increased level in its metabolites, due to the well established mechanism of antipsychotic drug action in their long-term administration (1). Based on the above-mentioned results, the sub-chronic administration of haloperidol (7 days) was considered. 

However, in this research we showed that in a sub-chronic study (for a 7 days period) only high doses of the compound 11b were able to modify the dopamine levels. It has been shown that normally COX-2 is expressed in low levels in nigral dopaminergic neurons, but it becomes up-regulated in both patients and experimental PD models (19). These suggest that different levels of COX-2 in normal and SNc lesioned rats may explain the aforementioned observations.

The effects of the compound 11b on other neurotransmitter systems (cholinergic-glutaminergic) have not been investigated and should be further studied in future works. 

## References

[1] Pemminati S, Nair V, Dorababu P, Gopalakrishna HN, Pai M (2007). Effect of ethanolic leaf extract of Ocimum sanctum on haloperidol-induced catalepsy in albino mice. Indian J. Pharmacol.

[2] Teismann P, Tieu K, Choi DK, Wu DC, Naini A, Hunot S (2003). Cyclooxygenase-2 is instrument in Parkinson’s Disease neurodegeneration. Proc. Natl. Acad. Sci. USA.

[3] McGeer PL, Schwab C, Parent A, Doudet D (2003). Presence of reactive microglia in monkey substantia nigra years after 1-methyl-4-phenyl-1, 2, 3, 6-tetrahydropyridine administration. Ann. Neurol.

[4] Fathi Moghaddam H, Shafiee Ardestani M, Saffari M, Navidpour L, Shafiee A, Rahmim A (2008). Dopaminergic but not glutamatergic neurotransmission is increased in the striatum after selective COX-2 inhibition in normal and hemiparkinsonian rats. Basic Clin. Pharmacol. Toxicol.

[5] Shafiee Ardestani M, Sadeghzadeh N, Mehrab H (2007). Effects of dexamethasone and betamethasone as COX-2 gene expression inhibitors on rigidity in a rat model of Parkinson’s disease. Indian J. Pharmacol.

[6] Akaike A, Kaneko S, Tamura Y, Nakata N, Shiomi H, Ushikubi F, Narumiya S (1994). Prostaglandin E2 protects cultured cortical neurons against N-methyl-D-aspartate receptor-mediated glutamate cytotoxicity. Brain Res.

[7] Katzung BG (2004). Basic and Clinical Pharmacology.

[8] Rall JM, Mach SA, Dash PK (2003). Intrahippocampal infusion cyclooxygenase-2 inhibitor attenuates memory acquisition in rats. Brain Res.

[9] Sharifzadeh M, Tavasoli M, Naghdi N, Ghanbari A, Amini M, Roghani A (2005). Post-training intrahippocampal infusion of nicotine prevents spatial memory retention deficits induced by the cyclo-oxygenase-2-specific inhibitor celecoxib in rats. J. Neurochem.

[10] Navidpour L, Shadnia H, Shafaroodi H, Amini M, Dehpour AR, Shafiee A (2007). Design, synthesis, and biological evaluation of substituted 2-alkylthio-1, 5- diarylimidazoles as selective COX-2 inhibitors. Bioorg. Med. Chem.

[11] Paxinos G, Watson C (1997). The Rat Brain in Stereotaxic Coordinates.

[12] Duran M, Alfonso B, Arias A (1998). Determination of biogenic amines in rat brain dialysates by high performance liquid chromatography. J. Liq. Chromotogr. Relat. Technol.

[13] Marchese G, Bartholini F, Ruiu S, Casti P, Saba P, Gessa GL, Gessa GL, Pani L (2002). Effect of the amisulpride isomers on rat catalepsy. Eur. J. Pharmacol.

[14] Kleven MS, Barret-Grévoz C, Slot LB, Newman-Tancredi A (2005). Novel antipsychotic agents with 5-HT1A agonist properties: Role of 5-HT1A receptor activation in attenuation of catalepsy induction in rats. Neuropharmacol.

[15] Silva SR, Futuro-Neto HA, Pires JG (1995). Effects of 5-HT3 receptor antagonists on neuroleptic-induced catalepsy in mice. Neuropharmacol.

[16] Pires JG, Costa PG, Saraiva FP, Bonikovski V, Futuro Neto HA (2003). Gender-related differences in the effects of nitric oxide donors on neuroleptic-induced catalepsy in mice. Braz. J. Med. Biol. Res.

[17] Fathi-Moghaddam H, Shafiee Ardestani M COX inhibition: Catalepsy and Striatum Dopaminergic-GABAergic-Glutamatergic Neurotransmission. Nature Precedings.

[18] Bishnoi M, Chopra K, Shrinivas K, Kulkarni SK (2007). Possible anti-oxidant and neuroprotective mechanisms of zolpidem in attenuating typical anti-psychotic-induced orofacial dyskinesia-A biochemical and neurochemical study. Progress Neuro-Psychopharmacol. Biol. Psychiatry.

[19] Singh NA, Singh A, Kulkarni SK (2002). Carvedilol attenuates neuroleptic-induced orofacial dyskinesia: possible antioxidant mechanisms. Br. J. Pharmacol.

[20] Singh NA, Kulkarni SK (2003). Quercetin, a bioflavonoid attenuated haloperidol induced orofacial dyskinesia. Neuropharmacol.

[21] Casper D, Yaparpalvi U, Rempel N, Werner P (2000). Ibuprofen protect dopaminergic neurons against glutamate toxicity in-vitro. Neurosci. Lett.

[22] Fathi-Moghaddam H, Shafiee Ardestani M (2008). Betamethasone can significantly decrease level of striatal glutamate in animal model of parkinson’s disease. Iranian J. Pharm. Res.

